# A Trust-Based Formal Model for Fault Detection in Wireless Sensor Networks

**DOI:** 10.3390/s19081916

**Published:** 2019-04-23

**Authors:** Na Wang, Jiacun Wang, Xuemin Chen

**Affiliations:** 1 Department of Computer and Information Engineering, Shanghai Polytechnic University, Shanghai 201209, China; 2 Department of Computer Science and Software Engineering, Monmouth University, West Long Branch, NJ 07764, USA; jwang@monmouth.edu; 3 Department of Engineering, Texas Southern University, Houston, TX 77004, USA

**Keywords:** formal model, fault detection, multi-factors, Petri nets, wireless sensor networks

## Abstract

Wireless Sensor Networks (WSNs) are prone to failures and malicious attacks. Trust evaluation is becoming a new method for fault detection in WSNs. In our previous work, a comprehensive trust model based on multi-factors was introduced for fault detection. This model was validated by simulating. However, it needs to be redeployed when adjustment to network parameters is made. To address the redeployment issue, we propose a Trust-based Formal Model (TFM) that can describe the fault detection process and check faults without simulating and running a WSN. This model derives from Petri nets with the characteristics of time, weight, and threshold. Basic structures of TFM are presented with which compound structures for general purposes can be built. The transition firing and marking updating rules are both defined for further system analysis. An efficient TFM analysis algorithm is developed for structured detection models. When trust factor values, firing time, weights, and thresholds are loaded, precise assessment of the node can be obtained. Finally, we implement TFM with the Generic Modeling Environment (GME). With an example, we illustrate that TFM can efficiently describe the fault detection process and specify faults in advance for WSNs.

## 1. Introduction

Wireless Sensor Networks (WSNs) consist of distributed sensors that can monitor the environment, communicate with each other, and transmit information. It can continuously and automatically monitor a given field or event without any human presence. The working process of the sensors is to take measurements of the surrounding environment and transmit data to a base station for further data processing. Currently, the applications of WSNs are popular in wide areas such as intelligent industry, health care monitoring, environment monitoring, home automation, smart transportation, natural disaster relief, etc.

Due to the inherent characteristics and natural environments, WSNs are prone to various types of attacks such as black hole attack, eavesdropping, etc. [1]. The emergence of new data handling technologies and analytic enabled the organization of big data in processes as an innovative aspect in WSNs. The big data paradigm, combined with the WSN technology, involves new challenges that are necessary to resolve in parallel [2]. Therefore, it is crucial to detect faults, which will enhance the overall performance by monitoring network activities, minimizing risk, and ensuring the network activities of the entity such as data gathering and data processing. In [3], fault diagnosis in WSNs through various fault detection algorithms was given. In [4,5,6,7,8,9,10], the authors proposed various fault detection techniques. These techniques focused on how to detect and deal with faults by deploying a real or virtual WSN. In order to get precise detection, the authors above proposed effective methods from different perspectives such as energy consumption and sensor circuits. However, in wireless sensor networks, the trust model has played an important role in identifying misbehaving nodes and providing collaboration among trustworthy nodes [11]. The authors of [12] proposed a protocol layer trust-based intrusion detection scheme for wireless sensor networks, and the work in [13] proposed an evaluation model and data fusion mechanism based on trust. They focused on different aspects to measure trust values. In [14,15], the authors showed that trust models can provide a metric for routing, aggregation, and faulty detection. In our former work [16], we introduced a comprehensive trust model, which is able to assess the trust values of nodes by private trust and interactive trust. We classified trust into two parts as interactive trust and private trust. Interactive trust describes the trust of a node’s interaction with its neighbor nodes based on interactive attributes. Private trust focuses on describing a node’s reputation based on private attributes. Both trust values are used for detecting abnormal events and faulty nodes. All of the methods above were proposed for fault detection focusing on trust assessment. However, they were all implemented by simulations, and each detection process was run on a real deployment. Therefore, adjustment may be needed when the detection result cannot meet the requirement. In order to avoid frequently employing WSNs, a formal model can help check the detection method instead of simulation.

Petri nets are a powerful formal approach in computer science and system engineering [17]. Petri nets combine a well-defined mathematical model with an intuitive graphical model. The theoretical aspect of Petri nets allows precise modeling and analysis of system behavior, while the graphical aspect enables visualization of the state changes of the modeled system. Petri nets are a superior choice for specifying the concurrency and competitiveness of systems. These advantages allow Petri nets to find application in various kinds of event-driven systems such as embedded systems, communication systems, manufacturing plants, networks, real-time computing systems, and so on. Timed Petri nets, in which event times are specified, are able to catch the time-related performance or real-time properties of a system [18]. With timed Petri nets, we can precisely specify the fault detection mechanism of complex wireless sensor networks.

In this paper, inspired by the modeling power of Petri nets, we propose a trust-based formal model (TFM) for fault detection in WSNs. Based on TFM, the process of fault detection is built. To demonstrate the effectiveness of TFM-based trust evaluation, we apply the analysis algorithm to our previous multi-factor trust model [16].

The rest of the paper is organized as follows. Related works are introduced in Section 2. The definitions of TFM is provided in Section 3. The analysis of TFM for the detection process is depicted in Section 4. The implementation of TFM is given in Section 5. The concluding remarks are drawn and future work is discussed in Section 6.

## 2. Related Works

There are several methods on different levels that can assess trust performances. They are paper proof, simulation, and formalization. The paper proof method, however, is prone to human error and is not scalable to deal with large systems [19]. Due to the inherent incompleteness of simulation coupled with the rounding errors of computers, results cannot be considered as 100% accurate, which is a serious limitation for WSNs [20]. Using rigorous mathematical techniques, formal methods can overcome the limitations of simulation and have been used to validate a wide range of hardware and software systems [21]. Formal methods have been explored for analyzing WSNs, but most of the existing work is focused on analyzing their functional aspects only. However, with the wide application of WSNs in safety and mission-critical domains, there is an emergent need to assess their performances accurately as well. Formal methods sometimes provide languages such as Z language with strict semantics and syntax, corresponding techniques for the construction of models of systems under development, and verification of these models against selected requirements [22]. As a consequence, quantitative and qualitative properties, such as trust value or event detection rate, can be evaluated.

Recently, a few formal methods have been used for event detection in sensor networks. Some methods utilized the traditional model to check and validate special aspects of WSNs. In [23], the authors performed the formal analysis of the optimal geographical density control algorithm in real-time Maude to verify the network coverage intensity and lifetime. Real-time Maude is a language and tool for specification and analysis in real-time and hybrid systems [24]. It is based on re-writing logic that can provide analysis ability, but cannot provide performance evaluation. The authors of [25] suggested the use of P-Maude to enhance probabilistic analysis. Probabilistic model checking methods and tools [26] such as Prism have also been used for probabilistic analysis of wireless systems. Nevertheless, the accuracy of probabilistic model checking is very limited for validating statistical properties. In [27], an algebraic approach to the fault detection for parabolic distributed parameter systems was described. The modulation functions approach was used to obtain an algebraic fault detection equation, which only depends on known signals and the fault.

In [28,29], the authors showed that most of the popular formal approaches are based on theoretical models such as finite state machine, timed automata and process algebra. Finite state machine approaches have been pointed out for the difficulty in dealing with hierarchical models. Timed automata are an extension of finite state machines by incorporating real-valued clocks. Many specification methods are based on timed automata, and one of the well-known ones is UPPAAL. However, timed automata are deterministic finite state machines, so they inherit the limitations of finite state machines such as state explosions in large and complex distributed systems. Several approaches based on process algebra and composition logic were presented [30]. However, these approaches were mainly developed for database systems, so the sensing activities and spatial and temporal properties of WSNs were not addressed.

Timed Petri nets are a powerful extension to regular Petri nets [18]. Petri nets have advantages to describe events in network applications. In [31], the authors proposed a Petri net-based approach for resource requirements analysis and introduced Resource-Oriented Workflow Nets (ROWN). Petri nets have graphical support for users to operate easily. If the models can be improved for complex attributes, Petri nets can be a powerful tool to evaluate the performances in WSNs. In [32], the authors proposed a fault detection method modeled with Partially-Observed Timed Hybrid Petri nets (POTHPNs). Discrete faults that affect continuous processes have been considered. The marking of some continuous places and the firing of some discrete transitions were assumed to be measured on-line. Abrupt faults were considered as unexpected firings of some discrete silent transitions. This method is suitable for the class of hybrid systems that concerns continuous processing driven by discrete controllers. In [33], the authors used stochastic Petri nets to build a hierarchical model for the trust evaluation. The model focused on the location and energy of nodes. In [34], the goal of the trust model was to provide the authenticity of public keys. The trust model presented in this paper is based on the modeling technique of colored Petri nets. Colored Petri nets are a compact description of regular Petri nets. They do not increase the modeling and analytic capability of Petri nets. Therefore, colored Petri nets are mainly used for reachability, deadlock, and invariants’ analysis.

In [35], based on Petri nets, the authors described a compact event description and analysis language for wireless sensor networks, namely MEDAL, for simultaneous monitoring of multiple events in a single network. MEDAL is a modified Petri net, which provides a more compact formal language for event description. It can capture the structural, spatial, and temporal properties of a complex event detection system, so as to assist system designers in identifying inconsistencies and potential problems. MEDAL is an improvement of formalization in WSNs, but it can only describe private attributes of events. In [36], we proposed a formal model for temporal-spatial event detection in Internet of Vehicles (IoV) based on Petri net. In IoV, the events are detected based on attributes such as location, speed, and arriving time. The model proposed in [36] focused on describing the relationship of location and time to get an event detection result.

## 3. A Trust-Based Formal Model

**Figure 1:**
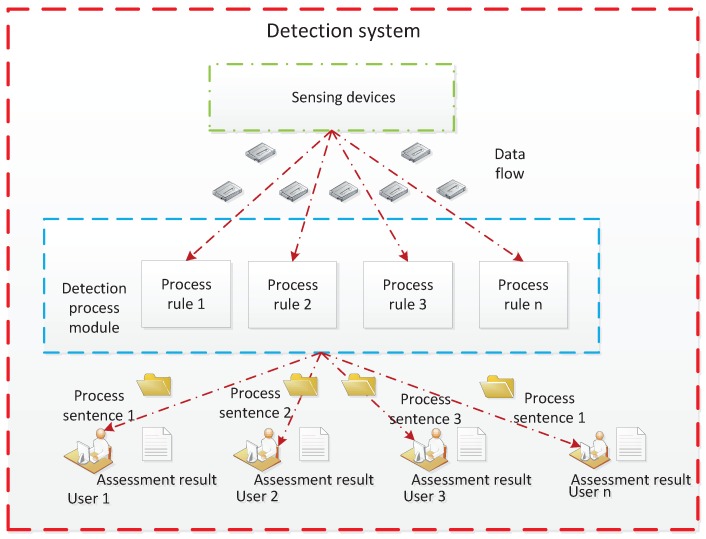
Detection system structure.

**Figure 2:**
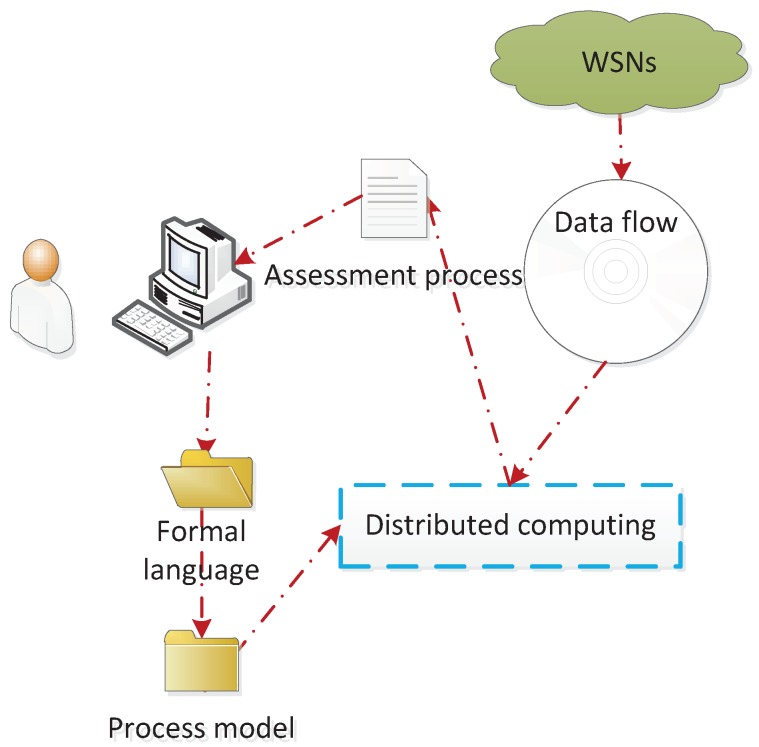
Detection process.

In WSNs, data collected by each sensor device are passed to the detection and processing module. This process is enforced during network operation by the processing program in a monitoring system. If the detection model can be validated in advance before being embedded into the system, the trial running cost of the system will be significantly reduced, and the accuracy of the detection will be improved. As shown in Figure 1, in a detection system, the user can describe the concerned event in formalized sentences and send it to the monitoring system. After receiving the formal statement, the monitoring system conveys it into the fault detection model. Then, the processing program generates the result based on the detection model, which will be fed back to the user. The detection process is described as in Figure 2.

Since most events in WSN applications are concurrent, asynchronous, distributed, and non-deterministic in nature, we use Petri nets as a base model to specify WSN operation. The basic structure of Petri nets consists of places (*P*), transitions (*T*), arcs, and tokens. In the graphic representation of Petri nets, circles represent states or conditions, dots are used to model instances or objects, rectangles model various kinds of actions, and arcs represent changes between states. When a token represents an object with a variety of attributes, the token has a value (color) that represents the specific characteristics of the object, such as a token representing a student (name, age, gender). For the sake of analysis, when time or latency needs to be modeled, each transition may have a time stamp that specifies the duration of its firing. Weights associated with Petri nets can also be set as the attribute of an arc. For specific applications, we can extend Petri nets into most functional ones. According to the requirements in WSNs, it is necessary to have a formal description of abstract items as follows:

- The time of data sensed
- The weight of each factor
- The threshold for decision

### 3.1. Definition of the TFM

Formally, a Petri net is defined as $PN=(P,T,I,O,M_{0})$ where

$P=\{p_{1},p_{2},\ldots p_{m}\}$ is a finite set of places;

$T=\{t_{1},t_{2},\ldots t_{n}\}$ is a finite set of transitions, P∪T≠∅,and P∩T≠∅;

I=T×P→N is an input function that defines directed arcs from places to transitions;

O=T×P→N is an output function that defines directed arcs from transitions to places;

$M_{0}=P\to N$ is the initial marking.

A marking in a Petri net is an assignment of tokens to the places of a Petri net. Tokens reside in the places of a Petri net. The number and location of tokens may change during the execution of a Petri net. The tokens are used to define the execution of a Petri net. A place containing one or more tokens is said to be marked [31].

The TFM can be described as an eight-tuple structure (P,T,I,O, $\;M_{0}$, μ,δ,θ) based on Petri nets, where P,T,I,O, and $\;M_{0}$ are classic definitions of Petri nets. In order to describe trust-based detection, we extend the basic Petri net with three items.

- μ is the weight on arcs, which represents the probability or importance of factors of a transition. μ:A→[0,1] and $\sum_{i=1}^{n}\mu_{i}=1$ where *n* is the number of input arcs into a transition. For example, if there is an arc (p,t), μ(p,t)=w means there is a probability of μ(p,t) inducing the token entering *t* from *p*. If the token has a capacity *c*, the new capacity will be c∗w.
- δ is a time guard for *T*, δ: *T*$\to [t_{1},t_{2}]$, and $t_{1}\leq t_{2}$. δ(T)=(a,b) means transition *T* can only fire during $t_{1}$ and $t_{2}$. Especially, if $t_{1}$ = $t_{2}$, that means the transition can only happen during $t_{1}$.
- θ is the threshold of token capacity in *P*, θ: P→R, and *R* is a real type data. θ(P) = $r_{2}$, means when the capacity of the token in *P* is greater than or equal to $r_{2}$, *P* can reach a new station.

Tokens are abstract representations of sensed data. When a transition fires, the values of a token will be updated according to the rules. In the TFM, the main data are the trust of different factors.

### 3.2. Trust Modeling

**Figure 3:**
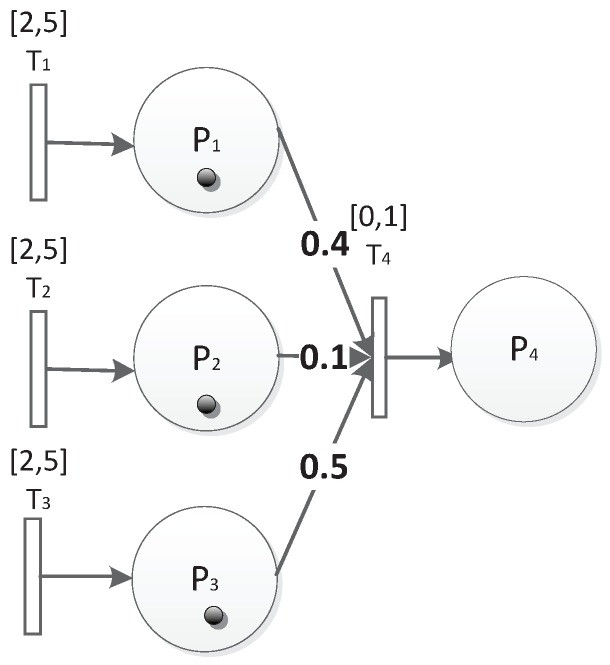
A simple Trust-based Formal Model (TFM).

There are several types of factors that can have an impact on trust in WSNs, and the value of each type of factor can be represented by a non-negative real number [16]. An evaluation process will consume factors for aggregating a new trust value. We use $F^{in}$ to describe the input factors consumed and use $F^{out}$ to describe the aggregation trust value. For an evaluation process $TP_{k}$, $F^{in}\left(TP_{k}\right)$ is associated with the input place and $F^{out}\left(TP_{k}\right)$ is associated with the output place. For example, considering Figure 3, assume $P_{4}$ is a state of aggregation output, and $P_{1}$, $P_{2}$, $P_{3}$ are states of input. When there is a process $TP_{4}$, which has three inputs, $F^{in}\left(TP_{4}\right)$ will be the factors’ values and $F^{out}\left(TP_{4}\right)$ will be the aggregation value of inputs according to special operations, which will be introduced later.

### 3.3. Rules in TFM

Rules for firing transitions are described as below:

A transition $T_{k}$ under the marking $M_{i}$ can be fired if and only if:

(1) $$t_{k}\in \delta \left(T_{K}\right)$$

(2) $$F^{in}\left(TP_{k}\right)>0$$

(3) $$F^{out}\left(TP_{k}\right)\geq \theta \left(P_{i}\right)$$

(4) $$M_{i}\geq I\left(t_{k}\right)$$

where $\delta (T_{k})$ is the valid sensing time in WSNs and $F^{in}\left(TP_{k}\right)$ is the current value of the token in the input place. Note that for a process $TP_{k}$, there may be more than one input. $F^{out}\left(TP_{k}\right)$ is the current value of the token in the output place, and θ is the threshold for entering $P_{i}$.

Condition (1) stands for time limit satisfaction; conditions (2) and (3) stand for valid value being available; and condition (4) stands for control readiness. These conditions must be met by trust factors simultaneously.

Rules for markings are described as below:

A token value may be changed when a transition fires, and it will be held in a new place due to the threshold.

(5) $$F^{out}\left(TP_{k}\right)=\sum_{j=0}^{n}\mu_{j}*F_{j}^{in}\left(TP_{k}\right)$$

where *n* is the number of factors.

Then, the new marking will be updated as below:

(6) $$M_{j+1}=M_{j}-I\left(T_{k}\right)+O\left(T_{k}\right)$$

### 3.4. An Example

In order to explain the TFM intuitively, we give an example here. Still considering Figure 3, it represents a system as follows:

$$\delta \left(T_{1}\right)=\delta \left(T_{2}\right)=\delta \left(T_{3}\right)=\left[2,5\right],\delta \left(T_{4}\right)=\left[0,1\right];$$

$$F_{3}^{in}\left(TP_{4}\right)=\left(0.9,0.09,0.8\right);$$

$$M_{0}=\left(1,1,1,0\right);$$

$$\mu \left(P_{1},T_{4}\right)=0.4,\mu \left(P_{2},T_{4}\right)=0.1,\mu \left(P_{3},T_{4}\right)=0.5;$$

$$\theta \left(P_{4}\right)=0.6,\theta \left(P_{1}\right)=\theta \left(P_{2}\right)=\theta \left(P_{3}\right)=0\left(default\right);$$

$T_{1}$, $T_{2}$, $T_{3}$ represent the three transitions that can fire after two and must fire before five time units. After the three transitions fire, new tokens are built. Since $P_{1}$, $P_{2}$, and $P_{3}$ receive tokens unconditionally, their thresholds are assigned as zero. Then, in one time unit duration, $T_{4}$ fires and $F^{out}\left(TP_{4}\right)=\sum_{i=1}^{3}\mu_{i}*F_{i}^{out}\left(TP_{4}\right)=0.774$. Since 0.774 is greater than the threshold of $P_{4}$, $P_{4}$ can be reached.

### 3.5. Structures of the TFM

**Figure 4:**
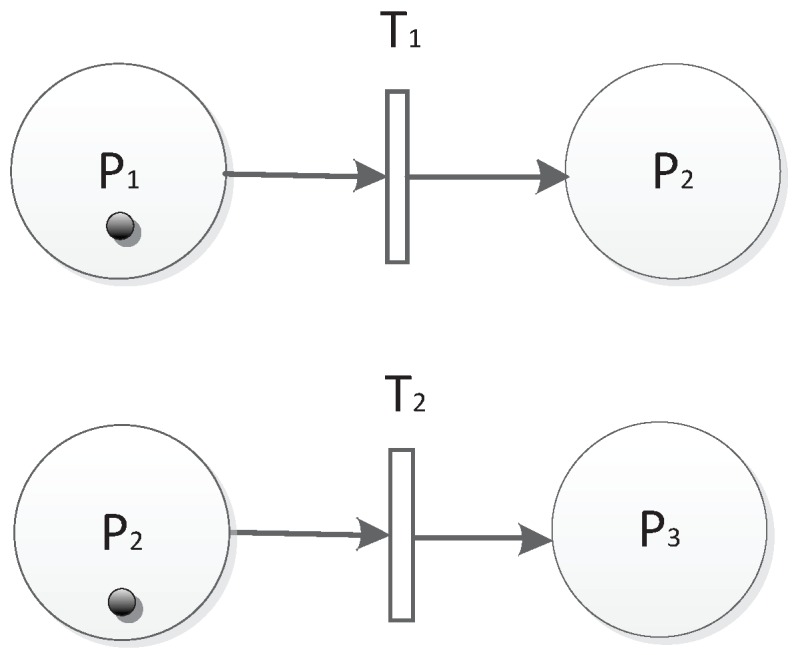
Two single logic units.

In order to evaluate a trust value based on multi-factors so as to assess the state of a node, we use two sequential places to model its evaluation process. One place stands for the sensing data, and the other stands for the assessment result. The evaluation action is modeled with a transition in between. The single logic unit is shown as in Figure 4.

**Figure 5:**
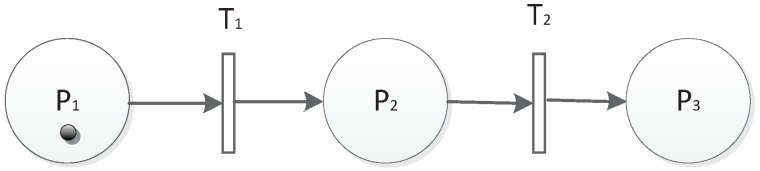
Sequential structure.

The sequential structure is shown in Figure 5. $P_{1}$ is the initial place; after $T_{1}$ is fired, $P_{2}$ is marked. If $T_{2}$ is fired, $P_{3}$ will be marked. In this case, $P_{2}$ is the shared place for $P_{1}$ and $P_{3}$. We describe the sequence as $T_{1}P_{2}\to T_{2}P_{3}$.

**Figure 6:**
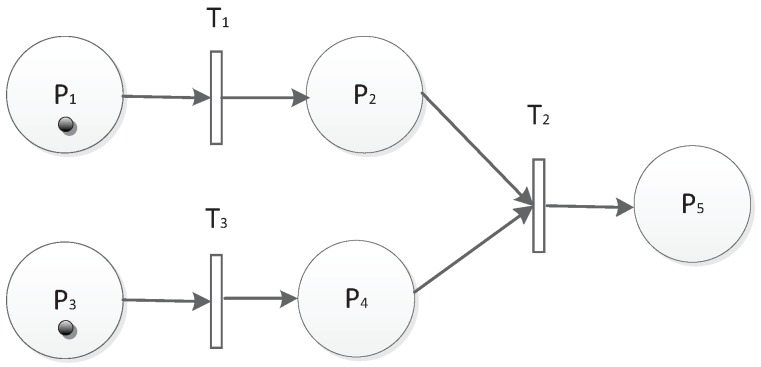
Parallel structure.

**Figure 7:**
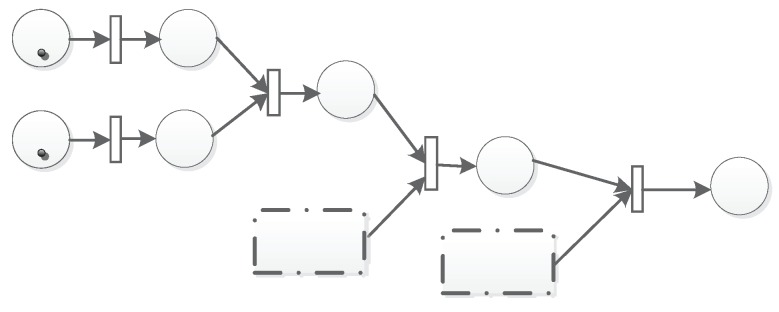
Chained parallel structure.

The second is the parallel structure, which is shown in Figure 6. There are two parallel units $T_{1}P_{2}$ and $T_{3}P_{4}$ that will fire $T_{3}$. $T_{2}$ can be fired if and only if there are tokens in both $P_{2}$ and $P_{4}$. Then, $P_{5}$ will be marked if the time and threshold conditions are also met. The parallel structure can be described as $T_{1}P_{2}\|T_{3}P_{4}$. The parallel structure can also be chained as shown in Figure 7.

**Figure 8:**
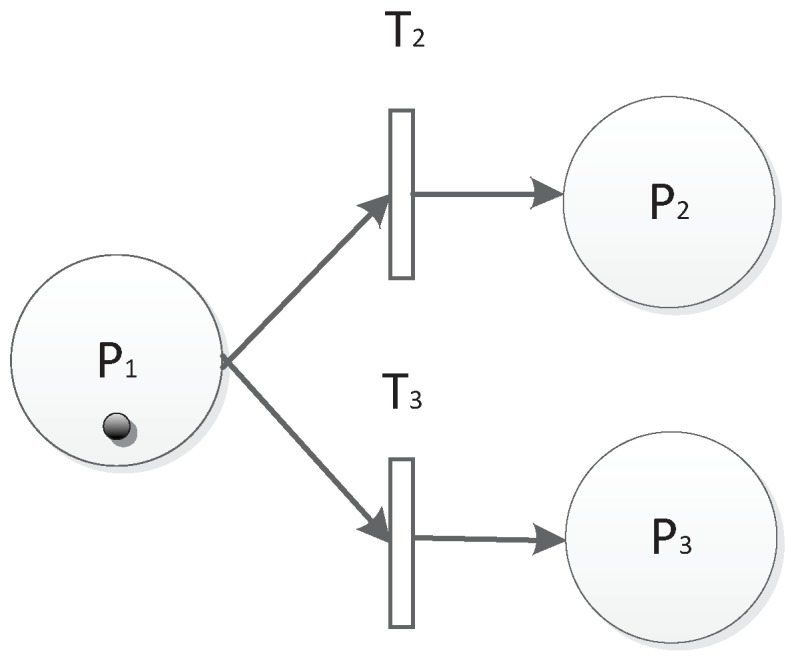
Choice structure.

**Figure 9:**
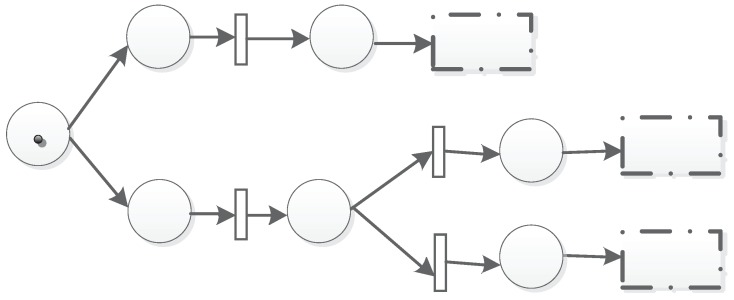
Chained choice structure.

The third is the choice of the structure that is shown in Figure 8. There are two choice units $T_{2}P_{2}$ and $T_{3}P_{3}$. Once there are tokens in $P_{1}$, $T_{2}$ or $T_{3}$ will be fired. Then, $P_{2}$ or $P_{3}$ will be marked if time and threshold conditions are also met. The choice structure can be described as $T_{2}P_{2}⊕T_{3}P_{3}$. The choice structure can also be chained as shown in Figure 9.

## 4. Analysis of the TFM

In our previous work [16], we defined a hierarchical network with a number of sensors. These sensors were placed in an area and transmit information within a certain radius. Each node maintained its identified number, sensing data, and location. There were three kinds of nodes including sink node, cluster heads, and member nodes. The member nodes sensed data and communicated with their heads directly. The cluster heads aggregated data sent by their member nodes and forwarded them to the sink node through other cluster heads by hops. The sink node is a central control node that can schedule the whole network. The running process of the network can be depicted as follows:

Event-driving stage: When there is a request for detecting in a certain field from the sink node, the sink node will send a sensing order to its neighbor nodes.

Self-organizing stage: The nodes receiving the request will act as the first-level cluster heads. The cluster heads will select their member nodes according to the cluster protocol.

Detecting stage: The member node senses data after receiving the request from its cluster head. The sensing action is frequent according to the sampling period.

Communication stage: The member nodes send data to the cluster head and its neighbors. In this stage, interaction and data aggregation are crucial to the trust value.

Data aggregation: After data transmitting, the cluster head has the information of its member nodes. In order to reduce information and energy consumption, data from the member nodes will be aggregated by the cluster head and be sent to the higher level cluster head.

Convergence stage: Data from different levels of cluster heads will be aggregated and sent hierarchically till the sink.

According to the behaviors of the nodes, we divided them into two types as normal nodes and outlier nodes. Normal nodes provided data that changed gradually and regularly. An outlier appeared to deviate markedly from other members in the same group [37]. Outlier nodes that did not perform normally were mainly caused by malicious activity, instrumentation error, human error, and a change in the environment [38]. In the network we have defined, the outlier nodes included faulty nodes providing fault data and event nodes providing event data that can reflect the change in the environment. In order to recognize normal nodes, faulty nodes and event nodes, we defined two types of trust: private trust and interactive trust. Private trust was mainly evaluated based on three factors:

(1) A node’s private trust in the last cycle.
(2) The number of times it deviated from the aggregation value in the current cycle.
(3) The number of times it sensed the same data consecutively.

It is clear that only the second factor is related to the aggregation data in a cluster. Therefore, even in such a cluster that a group of nodes has sensed firing and the others have not, the node that keeps its private trust above the normal threshold will be regarded as a normal one. If it does not, then it will be regarded as an outlier node. However, an outlier node may be either a faulty node or a node detecting an event such as firing. In order to decide whether the outlier node is a faulty node, we used interactive trust [16]. Interactive trust was mainly evaluated based on the data similarity and communication between a node and its neighbors. When the interactive trust of an outlier node is greater than event threshold, the outlier node should be recognized as an event node. Otherwise, the outlier node will be regarded as a faulty node [39].

**Figure 10:**
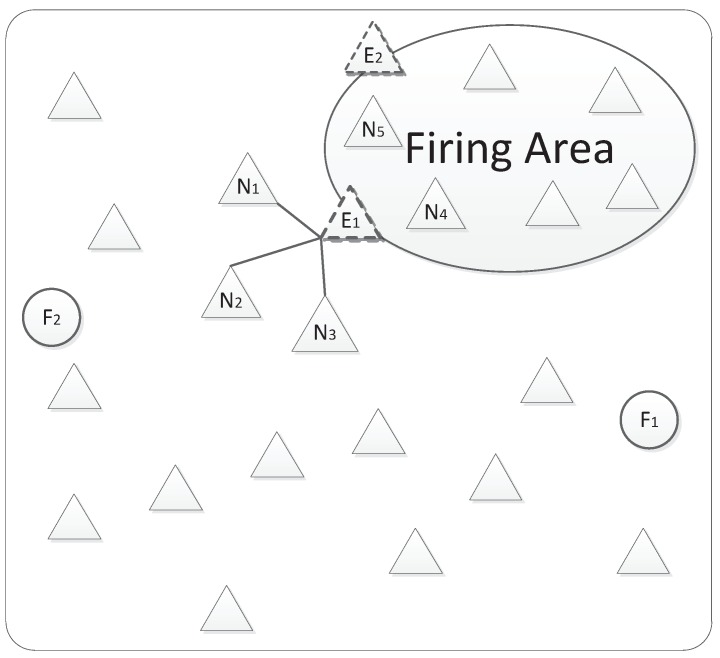
A distribution with four outliers $F_{1}$, $F_{2}$, $E_{1}$, and $E_{2}$.

For example, a set of nodes were deployed to monitor fire in a forest as shown in Figure 10. There were two faulty nodes $F_{1}$ and $F_{2}$. If there was no fire, normal sensors except $F_{1}$ and $F_{2}$ would keep a private trust greater than the normal threshold. Once there is a fire, the sensors in the fire area such as $N_{4}$ and $N_{5}$ would sense fire data. Meanwhile, the sensors on the fire border such as $E_{1}$ and $E_{2}$ would also sense fire data. However, the sensors far away from the fire area such as $N_{1}$, $N_{2}$, and $N_{3}$ would still sense normal data. If $E_{1}$ is in the same cluster with $N_{1}$, $N_{2}$, and $N_{3}$, but not in the same cluster with $N_{4}$ and $N_{5}$, its sensing data will deviate far from the aggregation value. If its private trust has not been remarkably reduced by the deviation, it will still be recognized as a normal one though its current sensing data are different from others’. If the deviation reduced its private value to under the normal threshold, it will be recognized as an outlier. Next, its interactive trust will be used to decide whether it is a faulty node or an event one. In this case, different from $F_{1}$ and $F_{2}$, $E_{1}$ and $E_{2}$ may be normal nodes or outlier nodes that just provide event data, but not faulty data.

**Figure 11:**
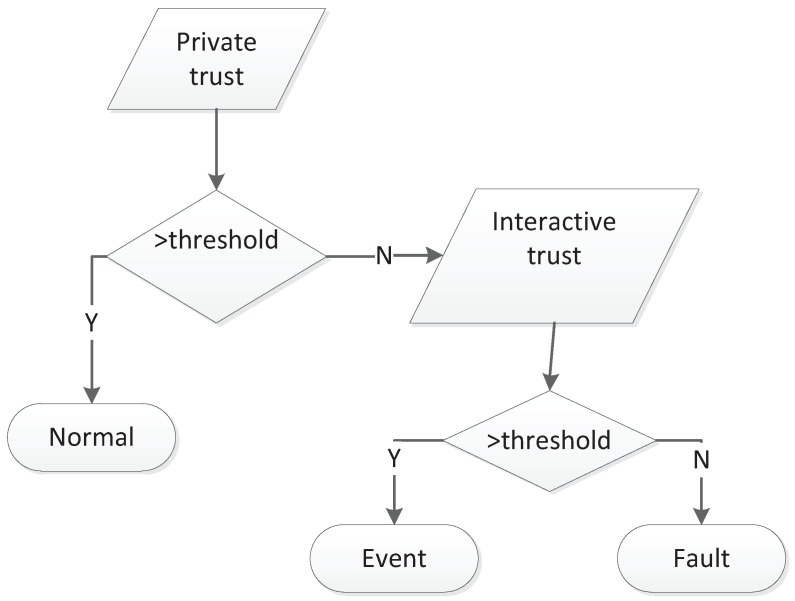
Fault detection based on trust [16].

In [16], as is shown in Table 1 and Table 2, interactive factors were abstracted as ITC (Interactive trust based on valid communication), ITD (Interactive trust based on data similarity) and ITT (Interactive trust based on clock synchronization), which are crucial when computing the trust value between nodes. Private factors depend on the node’s PTD (Private trust based on previous data), PTE (Private trust based on remaining energy), PTR (Private trust based on the misreading) and PTF (Private trust based on consecutive same sensing). The nodes must be penalized when sensing data deviate far from the aggregation value; they also should be awarded if sensing data are in normal distributed range consecutively. In order to depict the relations and importance of factors, we used a reciprocal matrix with a right characteristic root to calculate weight vectors. With interactive and private evaluations, we can detect fault nodes and event nodes, as shown in Figure 11. If the private trust value of a node is greater than the normal threshold, it will be regarded as being normal. Otherwise, if its interactive trust value is greater than the event threshold, it should be recognized as a event node. Otherwise, it will be regarded as a faulty node.

Using the TFM, we focus on assessing the status of nodes according to the time constraints and thresholds.

### 4.1. Analysis

With regard to time constraints, δ is used to control the transition duration to guarantee the validity of values in WSNs. If there is no requirement on the time limit, the default of δ is (−∞,+∞). For sequential and choice structures, the firing time of each transition must be in δ. For parallel structures, the firing role about time must be described specially.

Assume there are two transitions $T_{1}$ and $T_{2}$. The firing duration of $T_{1}$ is $\delta \left(T_{1}\right)=\left[t_{1},t_{2}\right]$, and for $T_{2}$, $\delta \left(T_{2}\right)=\left[t_{3},t_{4}\right]$. $T_{1}$ is enabled at time $\tau_{1}$ where $t_{1}\leq \tau_{1}\leq t_{2}$. If it fires at time $\tau_{1}+\phi_{1}$, according to the time rules in a timed Petri net, $\delta \left(T_{2}\right)=\left[max\left\{0,t_{3}-\phi_{1}\right\},t_{4}-\phi_{1}\right]$.

If there are more parallel transitions in a system, the firing time of a transition will shift for times according to the number of transitions firing before it, which is shown in (7). If we use *D* to denote the set of time constraints δ, then where $D=\{\delta \left(T_{j}\right),j=0\ldots n\}$, *n* is the number of places.

(7) $$\delta \left(T_{j}\right)=\left[max\left\{0,t_{j,1}-\sum_{i=2}^{j}\varphi_{i-1}\right\},t_{j,2}-\sum_{i=2}^{j}\varphi_{i-1}\right]$$

With regard to markings, consider a detecting procedure including $TP_{1},TP_{2},\ldots TP_{j}$. According to different structures, it can be described as:

If they are sequential:

(8) $$F^{out}\left(TP_{j}\right)=F^{out}\left(TP_{j-1}\right)*\mu_{j-1}$$

If they are parallel:

(9) $$F^{out}\left(TP_{j}\right)=\sum_{k=1}^{j-1}\mu_{k}*F^{out}\left(TP_{k}\right)$$

### 4.2. The TFM for the Trust Model Based on Multi-Factors

**Figure 12:**
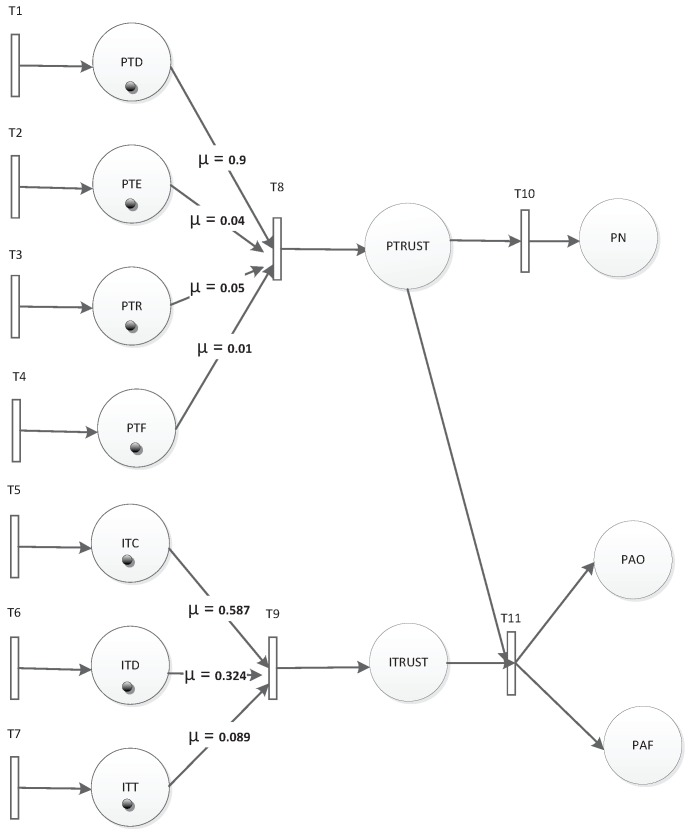
TFM for fault detection.

According to the logic description implied in Figure 11, when nodes are not regarded as normal, they may be outliers or abnormal nodes. If the interactive trust is greater than its threshold while private trust is less than its threshold, it may be the case that the node is located on the edge of the event area and detects an event. Otherwise, it can be treated as a fault node. To illustrate how to apply the TFM for fault detection, we built an example based on multi-factors, as shown in Figure 12. The numbers used in Figure 12 are defined in Table 3 and Table 4.

The trust model is described as follows:

P is the set for all places including factors and events. Factors represent the evaluation factors in the trust model; they are {PTD, PTE, PTR, PTF, ITC, ITD, ITT, PTRUST, ITRUST}. The first seven factors are explained in Table 1 and Table 2. PTRUST is a comprehensive trust deduced from PTD, PTE, PTR, and PTF which means private trust. ITRUST is a comprehensive trust deduced from ITC, ITD, and ITT, which means interactive trust. Events represent detection result; they are {PN, PAO, PAF}. PN represents a normal status of a node. PAO represents an abnormal status of a node, which is called an outlier. PAF represents an abnormal status of a node, which is called fault. We used interactive and private factors calculated by the method introduced in [16]. Current tokens are indicated in Table 3. In Table 3, there are three groups of factors, which are $Value^{1}$, $Value^{2}$, and $Value^{3}$. Different groups will get different assessments.

μ is the weight of each arc. If there are no branches, μ is one by default. Otherwise, μ is set according to the importance of a factor. In the trust model of [16], we used the reciprocal matrix with a right characteristic root to calculate weight vectors, as is shown in Table 4.

Since the data sensing and evaluation occurred periodically, we set $\delta (T_{i=1\ldots 4})$ for private factors’ relative transition, which can sense real-time factor values. Meanwhile, we set $\delta (T_{i=5\ldots 7})$ for interactive factors’ relative transition since interactive valuation will be executed in the case that private evaluation is less than the threshold. For other transitions, we left δ as (−∞,+∞), which means there were no time limitations for them. For this case, they are indicated in Table 5.

According to time constraint analysis, *D* will be:

$D_{0}=\left\{1\leq \delta \left(T_{1}\right)\leq 4,2\leq \delta \left(T_{2}\right)\leq 4,2\leq \delta \left(T_{3}\right)\leq 4,3\leq \delta \left(T_{4}\right)\leq 4\right\}$

$D_{1}=\left\{0.5\leq \delta \left(T_{2}\right)\leq 2.5,0.5\leq \delta \left(T_{3}\right)\leq 2.5,1.5\leq \delta \left(T_{4}\right)\leq 2.5\right\}$

$D_{2}=\left\{0\leq \delta \left(T_{2}\right)\leq 2,1\leq \delta \left(T_{4}\right)\leq 2\right\}$

$D_{3}=\left\{0\leq \delta \left(T_{4}\right)\leq 1\right\}$

$D_{4}=\left\{1\leq \delta \left(T_{8}\right)\leq 2\right\}$

$D_{5}=\left\{2\leq \delta \left(T_{5}\right)\leq 3,1\leq \delta \left(T_{6}\right)\leq 3,2\leq \delta \left(T_{7}\right)\leq 3\right\}$

$D_{6}=\left\{1\leq \delta \left(T_{5}\right)\leq 2,1\leq \delta \left(T_{7}\right)\leq 2\right\}$

$D_{7}=\left\{0\leq \delta \left(T_{7}\right)\leq 0.5\right\}$

$D_{8}=\left\{2\leq \delta \left(T_{9}\right)\leq 4\right\}$

$D_{9}=\left\{2\leq \delta \left(T_{1}0\right)\leq 4\right\}$

$D_{10}=\emptyset$

θ is the threshold that controls whether the token can enter place P. In a trust model, when the private trust reaches 0.8, a normal status is reached. Here, we set both θ(PN) = 0.8 and θ(PAO) = 0.8 according to our former work [16].

Using Algorithm 1, i.e., TFM analysis algorithm, we can evaluate the detection process. Suppose in the initial marking, weights and time constraints are set as shown in Table 3, Table 4 and Table 5, the attributes updating in processes of each branch reaching PN, PAO, and PAF are shown in Table 6.

In WSNs, it is important to detect fault nodes. In our previous trust model [16], we modeled the trust and evaluated the fault detection by simulation. Different from simulation, the analysis of the TFM does not need the detailed real-time data, but focuses on modeling time constraints and other limitations combining with trust values to determine the status of a node. Therefore, it is convenient for users to develop a detection method and validate its correctness by adjusting inputs. Furthermore, the detection rate based on the TFM can be achieved by building interpreters in the future.

**Algorithm 1** TFM analysis algorithm.**Input:**  **Input:**  Multi-factors;  Thresholds;  Weights;  Lower time;  Upper time; **Output:**  Node status;  j=0;  **while**$\left(M_{j}\right)$  **{**  for (i=0; i<n; i++)   {  **if**$\left(D_{i}\right)$  calculate $F^{out}\left(TP_{j}\right)$ using Equation (9);  }  calculate $F_{j+1}^{out}$ using Equation (8);  **if**
$\left(F_{j+1}^{in}\geq \theta_{j}\right)$  j++;  **}**

## 5. Implementation of the TFM

**Figure 13:**
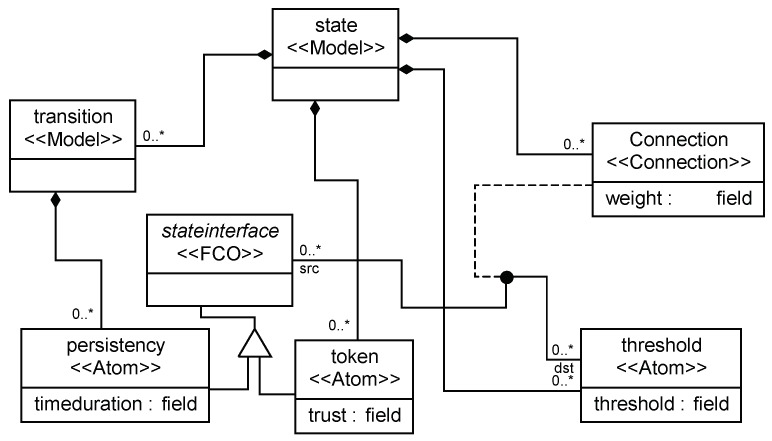
Model process for the TFM.

**Figure 14:**
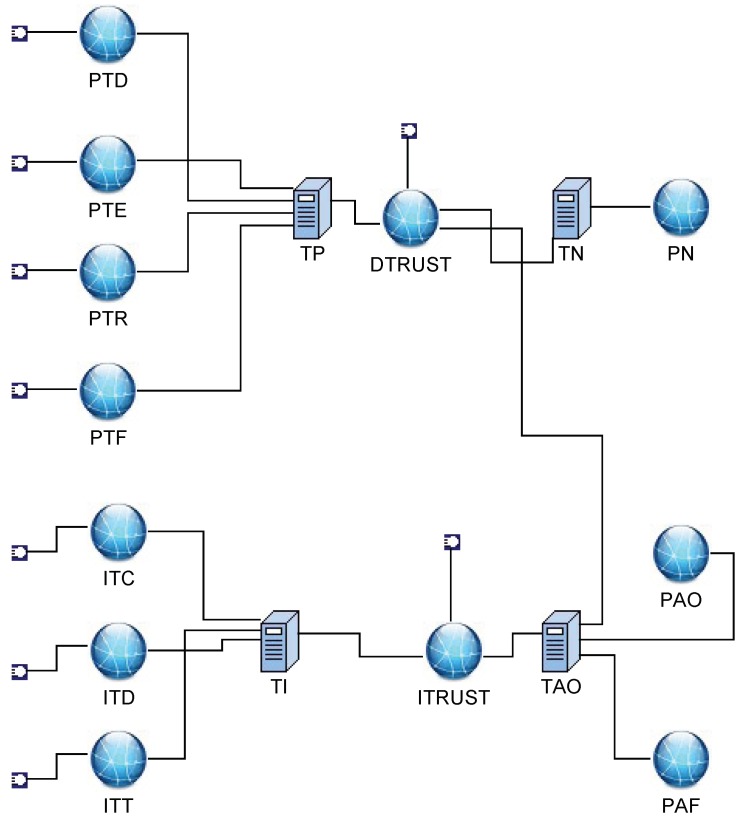
Model for trust in the TFM.

In order to provide a convenient environment for users, we used a Generic Modeling Environment (GME) as a platform to build the framework for the TFM applications, as shown in Figure 13. After the framework was built, registration and interpretation were executed. Then, different TFM applications can be constructed. The implementation part of the full environment is visualized for modeling and analysis in Figure 14.

For each place, there are properties such as token and threshold. For each transition, there are properties such as low time and high time. For each arc, there is a property named weight. We can set values for properties according to the TFM application requirements. The values can be modified easily on the property browser window. With model checking support, we can perform model checking for the TFM applications to validate detection methods, etc.

In our former work, we compared the trust model with a Trust Management Scheme (TMS) [39]. The result showed that the detection rate of our model was higher than TMS during the running time because we used private trust to confirm a fault node rapidly. However, the fluctuation of the detection rate was larger than TMS due to the temporary malicious judgment of event nodes.

**Figure 15:**
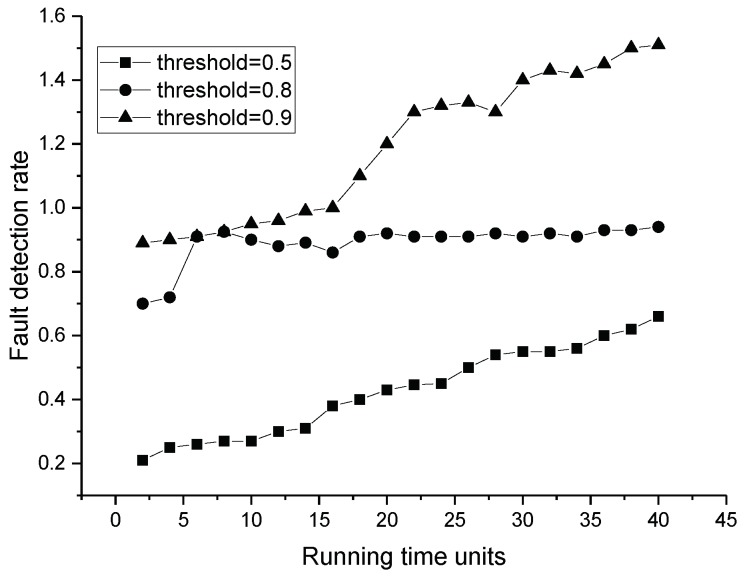
Fault detection rate with different thresholds.

**Figure 16:**
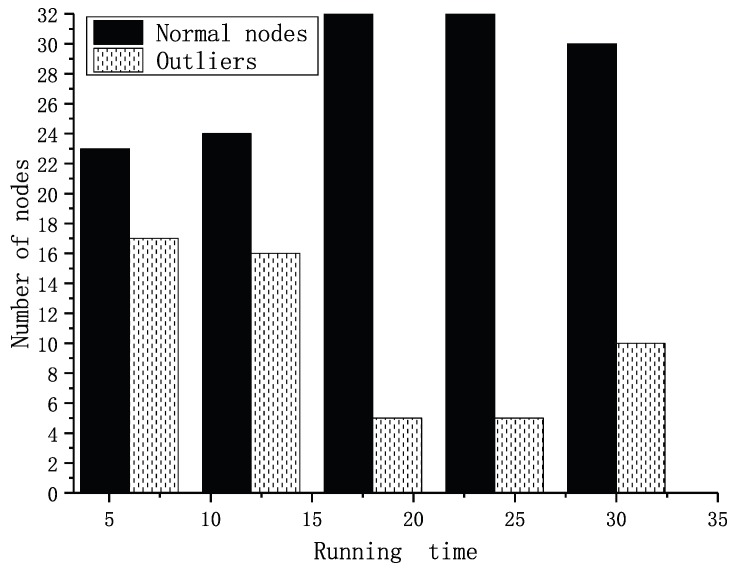
Nodes detected in our former work.

**Figure 17:**
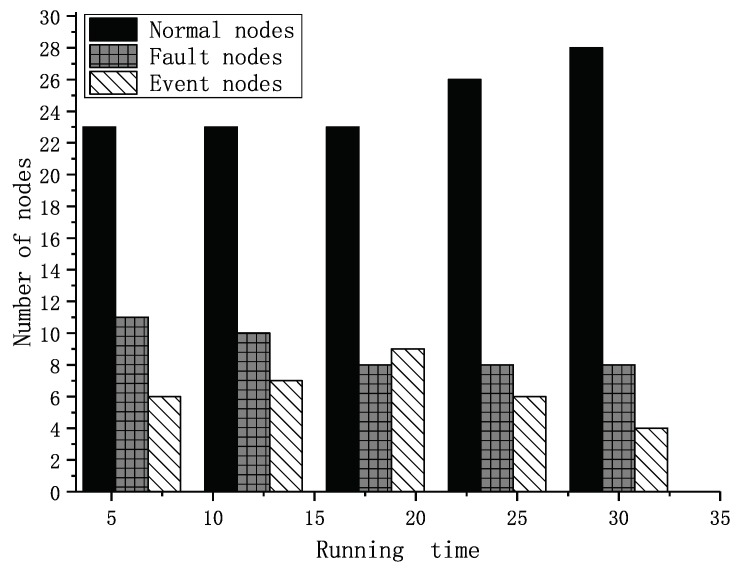
Nodes detected in the current work.

In the simulation, we tested forty sets of data that were the same as the former experiment. Each node had two to five neighbors in the experiment, and the node’s location was already known. The weights of factors were set as shown in Table 4 according to the reciprocal matrix in [16]. The thresholds for fault and event nodes were both set as 0.8 according to the statistics shown in Figure 15. In [16], we proved that the trust values were real numbers between zero and one. The trust values of normal nodes vibrated near 0.85. If the thresholds are set too low, some fault nodes cannot be detected. If the thresholds were set too high, some normal nodes may have been recognized as fault nodes. We assumed there was an event during the running time. Our former result is shown in Figure 16. It shows the numbers of different types of nodes including normal and fault ones. Within the running time, the normal ones will be detected more precisely, and the fault ones will be detected from outliers. Executing the TFM analysis algorithm, we evaluated both private trust values and the interactive trust value to detect whether the outlier was a fault or event node. The result in Figure 17 shows that the detecting of the normal nodes was stable, and the detecting of the event nodes from outliers was rapid.

**Figure 18:**
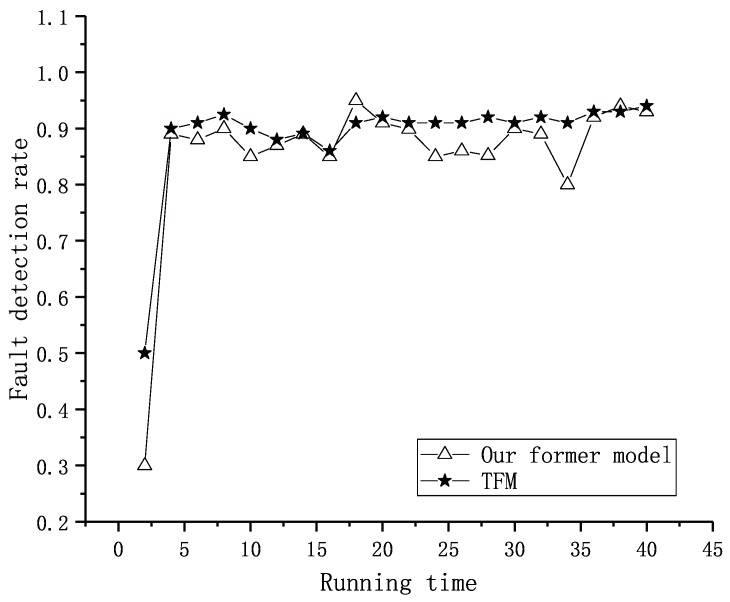
Comparison of the detection rate between two models.

Referring to Figure 12, there were three resulting states, PN, PAO and PAF. Transition TN related to PN had priority over TI related to PAO and PAF. When the token in state DTRUST was greater than 0.8, the node would be assessed as PN, which means normal. Otherwise, state ITRUST would be selected for assessment. When token in state ITRUST was greater than 0.8, the node would be assessed as PAO, which means event node. Otherwise, the node would be assessed as PAF, which means fault node. Once the event nodes had been recognized, the malicious judgment in our former model would decline. Then, the fault nodes could be excluded earlier, and the detection rate of fault nodes would become stable. The detection result comparison between this formal model and our former model is shown in Figure 18.

## 6. Conclusions and Future Work

In this paper, we used Petri nets to build a trust-based formal model that can describe the fault detection process. Compound model structures can be built from the basic TFM structures such as sequential, parallel, and choice structures. According to the TFM analysis algorithm, the fault detection process can be described by using the basic TFM structures, and the status of nodes can be assessed. With the implementation of the TFM, users can deploy a TFM application more visually and conveniently. The example and the result demonstrated that introducing time, weight, and threshold into Petri nets is suitable for the TFM. Once the places, thresholds, and transitions are set, the time constraints and the weights calculated from reciprocal matrix can make a precise evaluation of a node’s status so as to detect fault in advance.

In WSN applications, the TFM can be used to describe a system in a structured way. Meanwhile, the TFM allows users to modify existing designs quickly and re-evaluate updated designs conveniently without considering the network size.

However, we also need a software tool to process data from the model automatically. Typical processing tasks include running queries, generating program code, and building models automatically from information. The information may be provided by another data source (e.g., a database). In the future, we will develop a TFM interpreter to meet more application requirements. 

## Figures and Tables

**Table 1:** Interactive factors.

ITC	ITD	ITT
valid communication	data similarity	clock synchronization

**Table 2:** Private factors.

PTD	PTE	PTR	PTF
History data	Remaining energy	Penalty of misreading	Consecutive same sensing

**Table 3:** Initial marking in different cases.

Place	*Value* ^1^	*Value* ^2^	*Value* ^3^
PTD	0.89	0.29	0.29
PTE	0.93	0.93	0.93
PTR	0.7	0.7	0.7
PTF	0.88	0.88	0.88
ITC	0.94	0.94	0.64
ITD	0.82	0.82	0.82
ITT	0.77	0.77	0.77

**Table 4:** Weights of factors.

ITC	ITT	ITD	PTD	PTE	PTR	PTF
0.587	0.324	0.089	0.9	0.04	0.05	0.01

**Table 5:** The time for transition.

Transition	δ	Enabled Time	Firing Time	Firing Order
T1	[1, 5]	1	1.5	1
T2	[2, 4]	2	1	3
T3	[2, 5]	1	0.5	2
T4	[3, 6]	2	1	4
T5	[2, 3]	2	1.5	2
T6	[1, 4]	2	1	1
T7	[2, 4]	1	0.5	3
T8	[1, 2]	1	1	1
T9	[2, 4]	2	2.5	1
T10	[2, 4]	2	3	1

**Table 6:** Attributes in each branch.

	PN	PAO	PAF
D	{$D_{0},D_{1},D_{2},D_{3},$ $D_{4},D_{10}$}	{$D_{5},D_{6},D_{7},D_{8},$ $D_{10}$}	{$D_{5},D_{6},D_{7},D_{9},$ $D_{10}$}
M	$\left\{\begin{array}{c} (0.89,0.93,0.7,0.88,0.94,\\ 0.82,0.77,0,0,0,0,0),\\ (0,0,0,0,0.94,0.82,\\ 0.77,0.882,0,0,0,0),\\ (0,0,0,0,0.94,0.82,\\ 0.77,0,0.822,0,0,0) \end{array}\right\}$	$\left\{\begin{array}{c} (0.29,0.93,0.7,0.88,0.94,\\ 0.82,0.77,0,0,0,0,0),\\ (0,0,0,0,0.94,0.82,\\ 0.77,0.35,0,0,0,0),\\ (0,0,0,0,0,0,0,0.35,\\ 0.886,0,0,0),\\ (0,0,0,0,0,0,0,0.35,\\ 0,0,0.886,0) \end{array}\right\}$	$\left\{\begin{array}{c} (0.29,0.93,0.7,0.88,\\ 0.64,0.82,0.77,0,0,0,0,0),\\ (0,0,0,0,0.64,0.82,\\ 0.77,0.35,0,0,0,0),\\ (0,0,0,0,0,0,0,0.35,0,\\ 0.71,0,0),\\ (0,0,0,0,0,0,0,0.35,0,\\ 0,0,0,0.71) \end{array}\right\}$
$F^{out}$	$\left\{\begin{array}{c} 0.89*0.9+0.93*0.04+\\ 0.7*0.05+0.88*0.01=0.882,\\ 0.882*1=0.882 \end{array}\right\}$	$\left\{\begin{array}{c} 0.94*0.587+0.82*\\ 0.324+0.77*0.089=0.886,\\ 0.886*1=0.886,\\ 0.886*1=0.886 \end{array}\right\}$	$\left\{\begin{array}{c} 0.64*0.587+0.82*\\ 0.324+0.77*0.089=0.71,\\ 0.71*1=0.71,\\ 0.71*1=0.71 \end{array}\right\}$
θ	0.882> 0.8	0.886 > 0.8	0.71 < 0.8

## Abbreviations

The following abbreviations are used in this manuscript:

WSNs	Wireless Sensor Networks
TFM	Trust-based Formal Model
GME	Generic Modeling Environment
ROWN	Resource-Oriented Workflow Nets
